# Fiber Bragg Grating Dynamic Calibration Based on Online Sequential Extreme Learning Machine

**DOI:** 10.3390/s20071840

**Published:** 2020-03-26

**Authors:** Qiufeng Shang, Wenjie Qin

**Affiliations:** Department of Electronic and Communication Engineering, North China Electric Power University, No. 619 Yong Hua Street, Baoding 071003, China; baodingshang@ncepu.edu.cn

**Keywords:** optical fiber sensors, fiber Bragg gratings, online sequential extreme learning machine, dynamic calibration

## Abstract

The fiber Bragg grating (FBG) sensor calibration process is critical for optimizing performance. Real-time dynamic calibration is essential to improve the measured accuracy of the sensor. In this paper, we present a dynamic calibration method for FBG sensor temperature measurement, utilizing the online sequential extreme learning machine (OS-ELM). During the measurement process, the calibration model is continuously updated instead of retrained, which can reduce tedious calculations and improve the predictive speed. Polynomial fitting, a back propagation (BP) network, and a radial basis function (RBF) network were compared, and the results showed the dynamic method not only had a better generalization performance but also had a faster learning process. The dynamic calibration enabled the real-time measured data of the FBG sensor to input calibration models as online learning samples continuously, and could solve the insufficient coverage problem of static calibration training samples, so as to improve the long-term stability, accuracy of prediction, and generalization ability of the FBG sensor.

## 1. Introduction

Fiber Bragg grating (FBG) sensors have considerable advantages, such as high sensitivity, high accuracy, immunity to electromagnetic interference, stable chemical properties, compact size, and light weight. They are widely used in the measuring and monitoring of physical quantities, including strain, temperature, and humidity [1,2,3,4,5]. In recent decades, the development of optoelectronic technology has gradually expanded the application range of FBG sensors. FBG sensors currently find relevant applications in structural health monitoring [6,7], aeronautic prospecting [8], electric measurement [9], the production of medical devices [10,11], composite detection [12], and other fields. By monitoring the Bragg wavelength, it is possible to monitor the parameters that induce the wavelength shift of the FBG sensor, namely temperature and/or strain. The calibration is used to determine the mapping relationship between the wavelength and the physical quantity, and it is one of the critical factors affecting the performance of the sensor.

The static calibration of FBG sensor temperature measurement has been researched for a long time. As early as 1998, the authors of [13] pointed out that the Bragg wavelength of fiber gratings has a non-linear relationship with temperature over the range of 4.2–350 K, and determined the effect of embedding and the manufacturing process on the fibers’ temperature dependence, therefore it is essential to calibrate the measurement of fiber grating sensors. In 2006, the authors of [14] used a fifth-order polynomial to describe the temperature–wavelength correspondence and found that the wavelength drift caused by temperature change is highly non-linear over the range of 4.2–350 K. In 2012, the authors of [15] proposed a calibration algorithm based on a lookup table. The size of the lookup table can be selected according to the accuracy of the measurement data and the processing time requirements. The lookup table calibration algorithm reduces the processing time and measurement errors due to the imperfect fitting of polynomial functions when compared with polynomial fitting calibration. In addition, the authors of [16] and [17] put forward temperature calibration methods for FBG sensors based on a back propagation (BP) network and a radial basis function (RBF) network, respectively. They also found that neural networks have a higher calibration accuracy than polynomial fitting. The feasibility of neural networks was verified for complex calibration relationships. 

However, in actual engineering, we find that the wavelength–temperature response curve of an FBG sensor changes with time. This change is mainly caused by the temperature drift property of Fabry–Perot (F–P) etalons [18], the FBG pre-stretching amplitude, and the sealability between the FBG and the packaging material [19]. If the static calibration method is adopted, the measured error should be greatly increased. Therefore, we propose a dynamic calibration method that is based on an online sequential extreme learning machine (OS-ELM), which has the advantages of a fast learning speed, strong adaptability, and good generalization [20,21]. Additionally, it has been proven that an OS-ELM can be used in online prediction tasks in some fields [22,23,24]. To the best of our knowledge, this is the first study to report a long-term improvement in stability predictions for FBG dynamic calibration in the past ten years. This study may provide a new recognition of FBG sensors for measuring temperature.

## 2. Methods and Experiment Setup 

### 2.1. Extreme Learning Machine

The extreme learning machine (ELM) is the basis of an OS-ELM. The ELM is a single hidden layer forward neural network, including the input layer, hidden layer, and output layer. The N training samples and network’s output are described by $\left(x_{j},t_{j}\right)\in R^{n}\times R^{m},j=1,2,\cdots ,N$ and $f_{\tilde{N}}\left(x_{j}\right)=\sum_{i=1}^{\tilde{N}}\beta_{i}h_{i}\left(x_{j}\right),j=1,2,\cdots ,N$, respectively.

Here, $x_{j}$ is an n×1 input vector and $t_{j}$ is an m×1 target vector. $\tilde{N}$ is the number of hidden nodes, which is an approximation of N, $\beta_{i}$ is the weight between nodes $i^{\mathrm{th}}$ and the output layer. $h_{i}\left(x_{j}\right)$ is the output of the $i^{\mathrm{th}}$ node when input $x_{j}$,which is shown in Equation (1). $\partial_{i}$ is the weight between the input and node $i^{\mathrm{th}}$, $b_{i}$ is the bias of the $i^{\mathrm{th}}$ node.

(1) $$h_{i}\left(x_{j}\right)=g\left(\partial_{i}•x_{j}+b_{i}\right),j=1,2,\cdots ,N$$

According to [25], if $N=\tilde{N}$, then $\sum_{i=1}^{N}\beta_{i}h_{i}\left(x_{j}\right)=t_{j},j=1,2,\cdots ,N$, its matrix form is

(2) Hβ=T

The execution process of the ELM can be equivalent to the minimum norm of solving Equation (2), which means solving minimizing ‖Hβ−T‖. We assume that $\tilde{\beta }$ is the least square solution of Equation (2), it is obtained $\tilde{\beta }=H^{†}T$. Where $H^{†}$ is the Moore–Penrose generalized inverse of H, which can be solved by the orthogonalization method and the iterative method [25]. 

### 2.2. OS-ELM

The ELM is a static batch learning process. The training sample is not updated with the arrival of new data. The OS-ELM was proposed by G. Huang’s team to address this issue. The OS-ELM is generally divided into initial training and online learning. In the initial training phase, the network learns the initial $N_{0}$ training samples $\left(x_{j},t_{j}\right)\in R^{n}\times R^{m},j=1,2,\cdots ,N_{0}$. At the same time, $\beta_{0}$ is the solution to minimizing $\left\|H_{0}\beta -T_{0}\right\|$, where $\beta_{0}=K_{0}^{-1}H_{0}^{T}T_{0}$ and $K_{0}=H_{0}^{T}H_{0}$. When entering the online learning phase, the first new data or data block of size $N_{1}$ are newly learned, and the training sample is updated as $\left(x_{j},t_{j}\right)\in R^{n}\times R^{m},j=1,2,\cdots ,N_{0}+N_{1}$. At this point, the network is updated, shown in Equations (3) and (4).

(3) $$\beta_{1}=K_{1}^{-1}{\left[\begin{array}{c} H_{0}\\ H_{1} \end{array}\right]}^{T}\left[\begin{array}{c} T_{0}\\ T_{1} \end{array}\right]$$

(4) $$K_{1}={\left[\begin{array}{c} H_{0}\\ H_{1} \end{array}\right]}^{T}\left[\begin{array}{c} H_{0}\\ H_{1} \end{array}\right]$$

To infer the characteristics of continuous online learning, considering the relationship between $\beta_{0}$ and $\beta_{1}$, leads to

(5) $$\beta_{1}=\beta_{0}+K_{1}^{-1}H_{1}^{T}\left(T_{1}-H_{1}\beta_{0}\right)$$

Pushing to generalization, when learning the $i^{\mathrm{th}}$ data or data block, there are

(6) $$\beta_{k+1}=\beta_{k}+K_{k+1}^{-1}H_{k+1}^{T}\left(T_{k+1}-H_{k+1}\beta_{k}\right)$$

(7) $$K_{k+1}=K_{k}+H_{k+1}^{T}H_{k+1}$$

### 2.3. Experiment Setup

**Figure 1:**
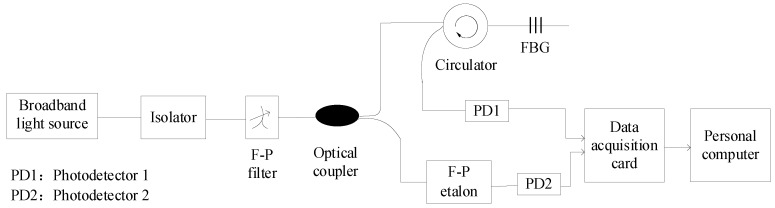
The fiber Bragg grating (FBG) sensing system.

In this paper, the experiment setup was as depicted in Figure 1. The temperature change can be detected by measuring the wavelength shift of FBG. As shown in Figure 1, line segments without arrows represent optical transmission, while those with arrows represent electrical transmission. The light from the broadband light source passed through the isolator. An F–P filter with a center wavelength of 1550 nm, a free spectral range (FSR) of 98.8 nm, and a bandwidth of 0.177 nm was adopted in this system. The tunable F–P filter was utilized to obtain a narrow-band tunable light, which scanned broadband light under the driving of a triangle wave. The narrow-band tunable light was split into two branches using an optical coupler. The upper branch was transmitted to the FBG through the circulator, and the reflected light was detected by the photodetector (PD1). When the transmission wavelength of the tunable F–P filter coincided with the reflection wavelength of the FBG, the PD1 detected the maximum light intensity. The lower branch into the F–P etalon was detected by another photodetector (PD2). The F–P etalon was similar in structure to the F–P filter, and its main part was also composed of an F–P cavity. The F–P etalon which had an FSR of 0.798 nm and a fineness of 6.61, was selected with a wavelength marking function as a wavelength reference. PD1 and PD2 had an operating wavelength range of 1100–1650 nm, a bandwidth of 4 MHz, a dark current of less than 0.85 nA, and a sensitivity of −52 dBm. PD1 and PD2 converted the detected optical signal into an electrical signal, then the electrical signal was sent to a Personal Computer (PC) via a data acquisition card, and then the PC performed denoising and peak detection. The F–P etalon was used as the wavelength reference to calculate the Bragg wavelength of the FBG. In this paper, the data acquisition card was used to simultaneously acquire the FBG reflection spectrum and the transmission spectrum of the F–P etalon, since the wavelength value of each positive peak in the transmission spectrum of the F–P etalon was known. Therefore, the Bragg wavelength of the FBG was determined by comparing the peak position of the FBG reflection spectrum with that of the F–P etalon transmission spectrum.

## 3. Results and Discussion

### 3.1. Data Set

**Figure 2:**
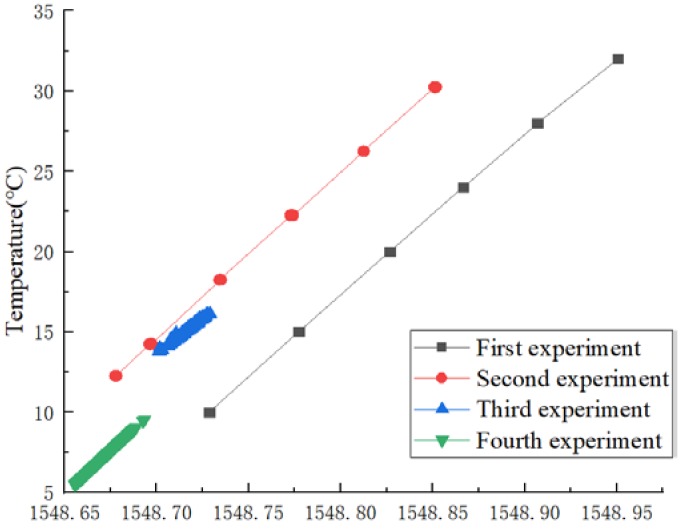
The four data acquisition experiments.

In order to verify the improvement in the measurement accuracy, generalization ability, and long-term stability of an OS-ELM for FBG sensor dynamic calibration, four data acquisition experiments were conducted, respectively, and wavelength–temperature pairs were provided. The experimental results are displayed in Figure 2. The four experiments were conducted in chronological order, with an interval of five months between the first experiment and the second experiment, five days between the second experiment and the third experiment, and nine months between the third experiment and the fourth experiment. In the first experiment, six temperatures were taken: 10, 15, 20, 24, 28, and 32 °C. The second experiment also took six temperatures, unevenly: 12, 14, 18, 22, 26, and 30 °C. The ranges of the third and fourth experiments were (13–16 °C) and (5–9 °C), respectively. It can be seen from Figure 2 that the FBG wavelength and temperature maps of the four experiments are different curves, and it is impossible to fit a single curve to represent their relationship. The discrepancy between measurement sets was mainly caused by the temperature drift property of the F–P etalon, the FBG pre-stretching amplitude, and the sealability between the FBG and the packaging material. Since eliminating the discrepancy from the optical path with hardware would increase the complexity and cost of the system, this paper studies a method of dynamic calibration to eliminate the discrepancy.

### 3.2. Simulated Analysis

**Figure 3:**
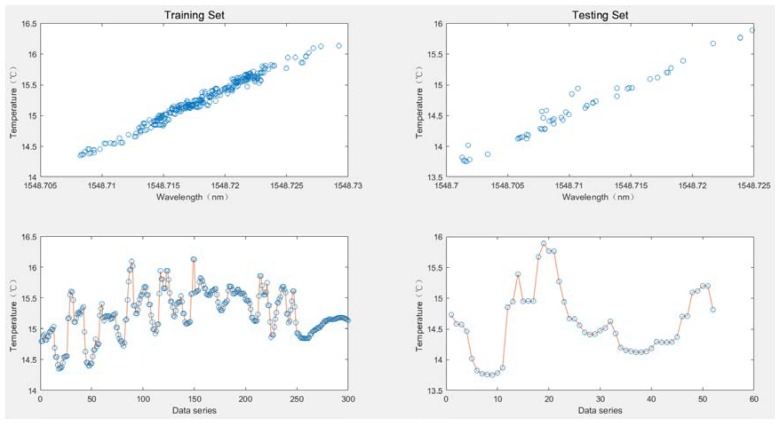
Data for the prediction accuracy experiments.

**Figure 4:**
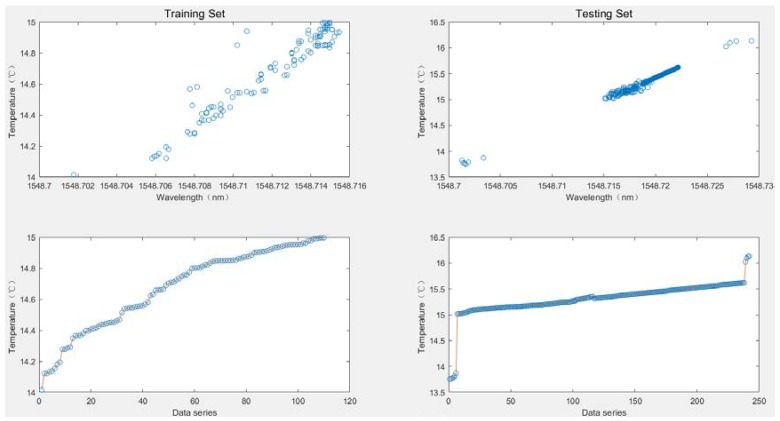
Data for the generalization ability experiments.

When comparing the performance of the dynamic calibration model with other static calibration models, an ELM was employed as the static model of an OS-ELM to compare with other calibration models for better control variables. Due to the limited space of the article, 352 pairs of data from the third experiment with the most severe noise among the four experiments were taken for verification. The 352 pairs of data were first randomly divided into 300 pairs as a training data set, and the remaining 52 pairs as a testing data set for accuracy testing, as shown in Figure 3. Figure 4 gives the data set for generalized performance testing. A total of 110 data points in the temperature range (14–15 °C) were taken as the training data set, and the remaining 242 data points were used as the testing data set.

The ELM commonly used activation functions that have a sigmoidal function (sig), a sine function (sin), a hardlim function (hardlim), and a radial basis function (radbas). The ELM model performs differently when different activation functions are used. The performances of these four activation functions were compared in terms of the root mean square error (RMSE) and goodness of fit ($R^{2}$) given by Equations (8) and (9), respectively, and the results are shown in Table 1.

(8) $$RMSE=\sqrt{\frac{1}{N}\sum_{i=1}^{N}{\left(f_{\tilde{N}}\left(x_{j}\right)-t_{j}\right)}^{2}}$$

(9) $$R^{2}=\frac{{\sum_{i=1}^{N}\left(t_{i}-\bar{f_{\tilde{N}}\left(x_{j}\right)}\right)}^{2}}{{\sum_{i=1}^{N}\left(f_{\tilde{N}}\left(x_{j}\right)-\bar{f_{\tilde{N}}\left(x_{j}\right)}\right)}^{2}}$$

where N is the total number of testing samples, $\tilde{N}$ is the number of hidden layer neurons. $f_{\tilde{N}}\left(x_{j}\right)$ and $t_{j}$ are the temperature measurement from the ELM and thermometer, respectively. $\bar{f_{\tilde{N}}\left(x_{j}\right)}$ is the mean of $f_{\tilde{N}}\left(x_{j}\right)$.

Because the RMSE described the precision of the prediction, its value was close to 0, which means a better prediction performance. Nevertheless, the closer the $R^{2}$ value is to 1, the better the fitting degree of the observed regression curve. As shown in Table 1, it is evident that the hardlim function had the worst performance. The sigmoid function returned the smallest value of RMSE, and the sine function received the shortest training time. The result of using a radial basis function is close to the sigmoid function in the evaluation criterion of $R^{2}$. However, the RMSE of the sigmoid function was smaller than that of the radial basis function. A comprehensive analysis showed that the ELM performs best when using a sigmoid function.

The prediction accuracy of the ELM, polynomial, BP, and RBF were also compared. In order to make the comparison fair, the results with the best performance in each calibration model were compared. As shown in Table 2, the polynomial took the least time, the RMSE for the ELM was the lowest, and $R^{2}$ for all models was very close. In terms of real-time, the polynomial was the best, and ELM was the best in terms of accuracy. As a prediction model, the generalization performance should also be considered. The generalization performance of each model is compared below.

**Figure 5:**
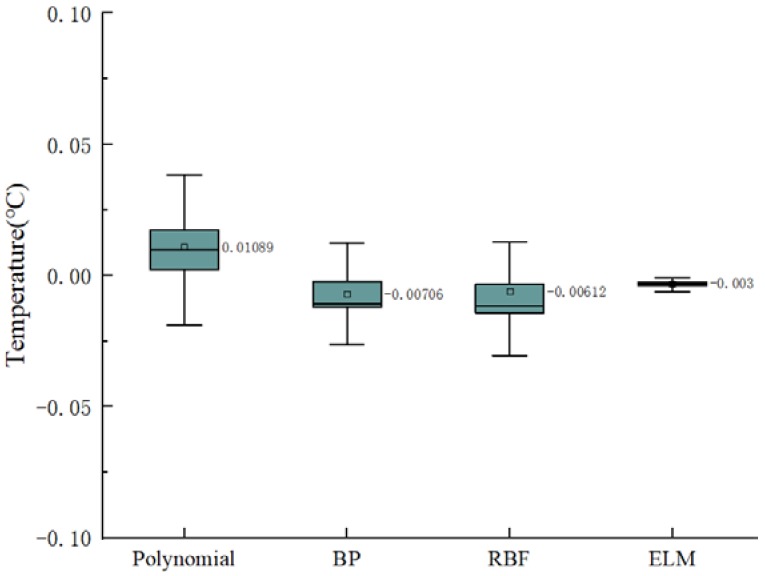
A boxplot of prediction errors for different calibration models.

Table 3 compares the generalization performance of the polynomial, BP, RBF, and ELM. The RBF and ELM performed best in RMSE and $R^{2}$, but the RBF took more time than the ELM. Figure 5 shows the boxplot of the differences between the predicted and observed values of the four models to analyze their stability. It can be seen from Figure 5 that the generalization prediction error of the polynomial was the largest, while the error of the ELM was the smallest. Meanwhile, the line extending from both sides of the ELM box is the shortest in the boxplot, so the ELM prediction was more stable than the others.

By comparing the prediction accuracy and generalization performance of the four models, including the polynomial, BP, RBF, and ELM, it was found that the prediction accuracy and generalization performance of the ELM model was better than that of the other three models.

### 3.3. Dynamic Calibration

The ELM model can be considered as a model in which the OS-ELM only has initial training, so the ELM is a static model. The calibration based on the OS-ELM is dynamic, and the calibration model can be continuously updated as new data arrives, rather than retraining the model. The dynamic calibration of the performances of the two aspects of stability and generalization was evaluated. The long-term stability was verified by the first and third experimental data (interval of five months), and the short-term stability was verified with the second and third experimental data (interval of five days). Date from the first experiment (10–32 °C) and data from the fourth experiment (5–9 °C) (interval of 14 months) verified the long-term generalization performance.

**Figure 6:**
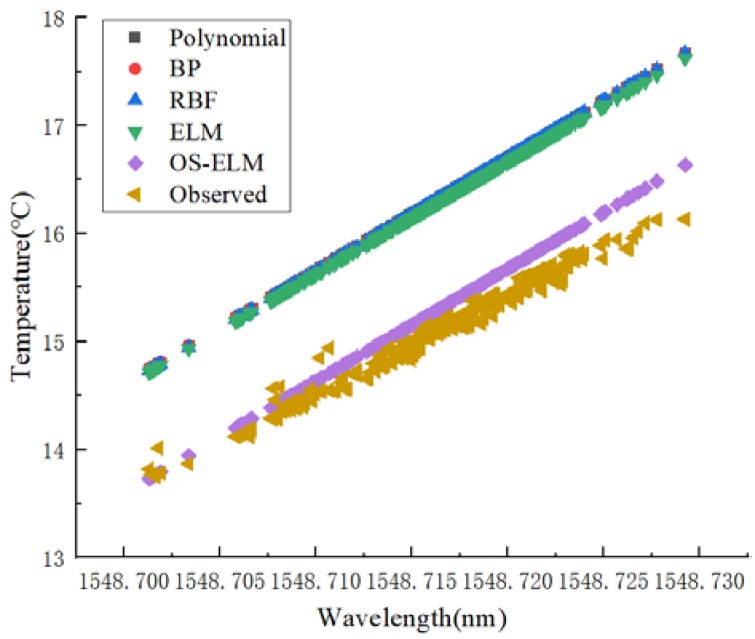
Performance comparisons of different calibration models in terms of short-term stability.

**Figure 7:**
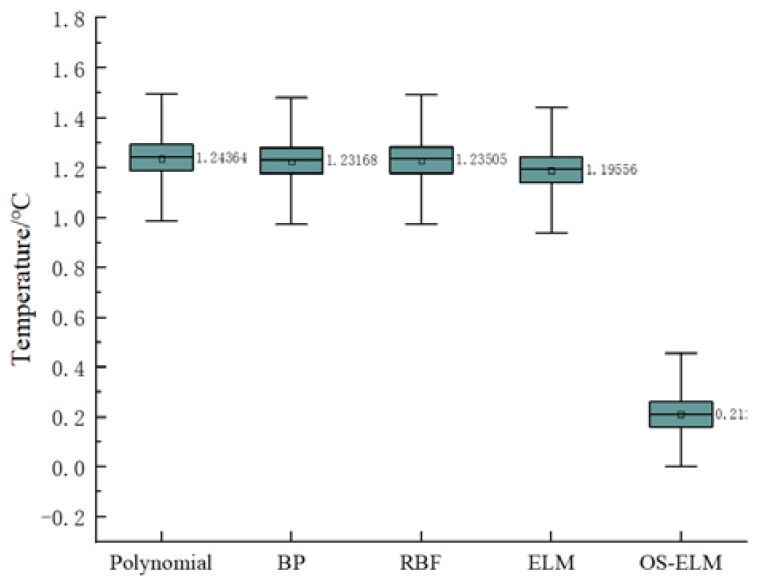
A boxplot of prediction errors for different calibration models in terms of short-term stability.

Firstly, the short-term stability was studied. The data set of the second experiment was used to train and calibrate the network. Then the trained network was used to predict the data of the third calibration experiment. The prediction results are shown in Figure 6, and the prediction error boxplot is shown in Figure 7. The polynomial, BP, RBF, and ELM prediction errors were 1.2436 °C, 1.2316 °C, 1.2350 °C, and 1.1956 °C, respectively, and the OS-ELM prediction error was 0.2 °C. 

**Figure 8:**
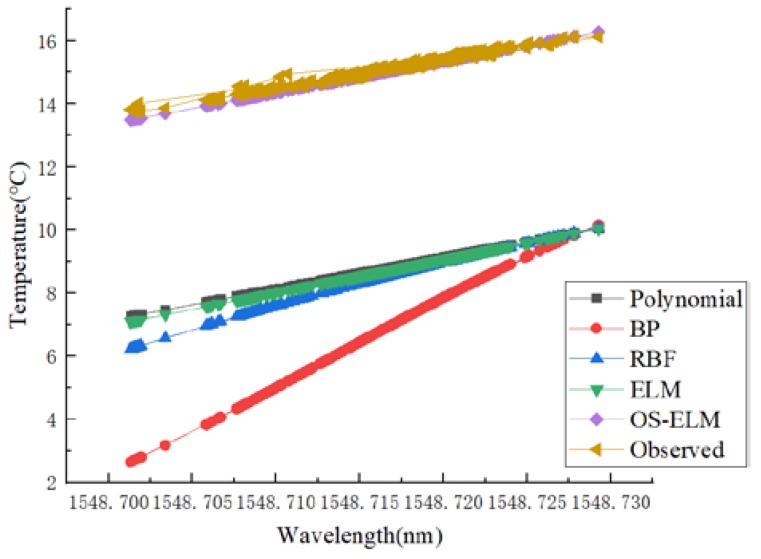
Performance comparisons of different calibration models in terms of long-term stability.

**Figure 9:**
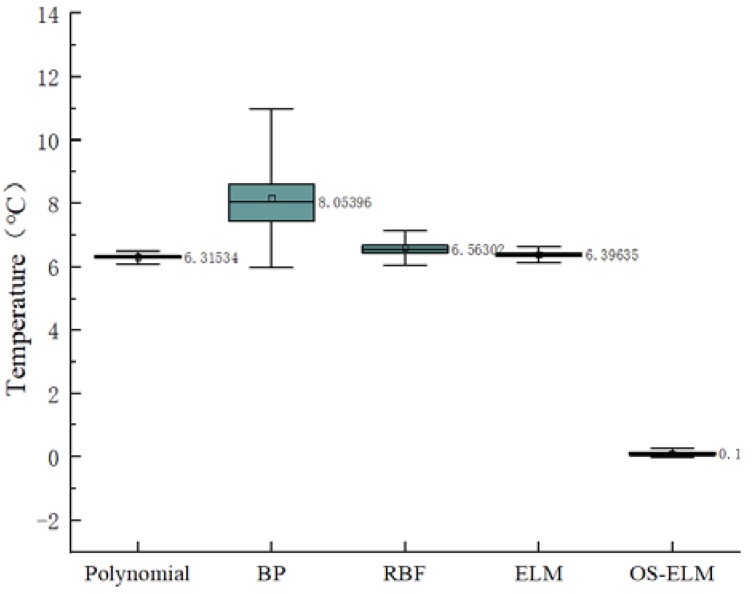
A boxplot of prediction errors for different calibration models in terms of long-term stability.

When verifying long-term stability, the data set of the first experiment was used to train and calibrate the network, and then the data of the third experiment were predicted by the trained network. The prediction results are shown in Figure 8, and the prediction error boxplot is shown in Figure 9. The advantages of the OS-ELM dynamic calibration in predicting accuracy and stability can clearly be observed.

**Figure 10:**
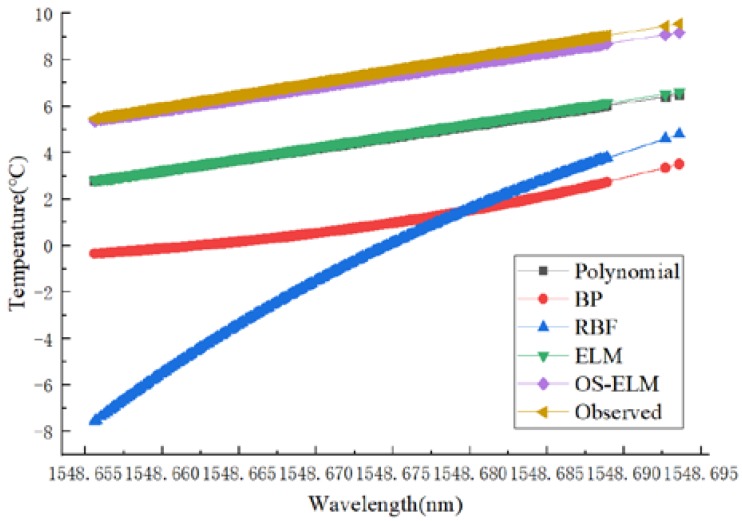
Performance comparisons of different calibration models in terms of generalization.

**Figure 11:**
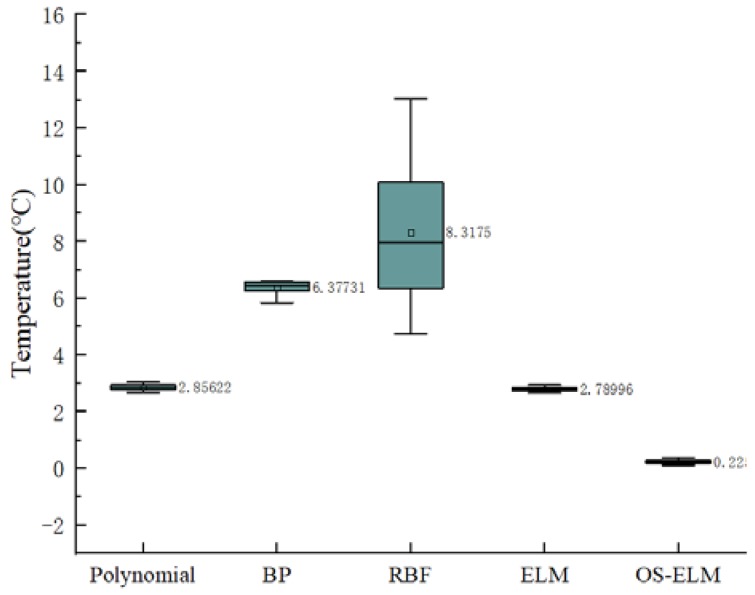
A boxplot of prediction errors for different calibration models in terms of generalization.

In order to study the long-term generalization performance of dynamic calibration, the data set of the first experiment was used to train the calibration network, and then the trained network was used to predict the data of the fourth experiment. The online learning sample was the data of the fourth experiment in the range of (5–6 °C). The prediction results are shown in Figure 10, and the prediction errors boxplot is shown in Figure 11. The apparent advantages of the OS-ELM can also be seen.

The comparative analyses above show that the dynamic calibration model based on the OS-ELM not only has an excellent generalization performance but also has a high prediction accuracy. The dynamic calibration can realize the sensor field-measured data and continuously input it into the network model as the online learning sample, which solves the problem of large drift errors of the static calibration model and insufficient coverage of the initial training sample.

## 4. Discussion

This paper provides a new dynamic model updating method, which is different from the traditional static calibration. In the dynamic updating phase, both the current prediction accuracy and the historical record are considered, which helps to reduce the fitting error of insufficient online learning samples. Besides, the dynamic calibration based on the OS-ELM significantly improved the prediction accuracy and generalization performance compared with previous static calibration methods. The maximum absolute error of the short-term stability experiment was 0.502 °C, that of the long-term stability experiment was 0.516 °C, and that of the long-term generalization experiment was 0.374 °C. Future research will focus on improving the calibration model according to the data characteristics.

## Figures and Tables

**Table 1:** Performance of the prediction accuracy of different activation functions.

Types of Activation Function	Time (s)		RMSE (°C)		$R^{2}$	
	Training	Testing	Training	Testing	Training	Testing
sig	0.1094	0	**0.0533**	**0.1027**	0.9772	**0.9698**
sin	**0.0154**	0	0.0555	0.1803	**0.9790**	0.8983
hardlim	0.0156	0	0.3677	0.8426	−5.5816 × 10^−12^	3.0073 × 10^−13^
radbas	0.0625	0	0.3774	0.8660	0.9772	**0.9698**

**Table 2:** Performances of prediction accuracy of different calibration models.

Types of Calibration Model	Time (s)		RMSE (°C)		$R^{2}$	
	Training	Testing	Training	Testing	Training	Testing
Polynomial	**0.0156**	**0**	0.0544	0.1327	0.9781	0.9686
BP	0.4063	0.0156	0.0535	0.1601	**0.9789**	0.9220
RBF	7.9688	0.0544	0.0625	0.1321	0.9781	0.9686
ELM	0.1094	**0**	**0.0533**	**0.1027**	0.9772	**0.9698**

**Table 3:** Performances of generalization of different calibration models.

Types of Calibration Model	Time (s)		RMSE (°C)		$R^{2}$	
	Training	Testing	Training	Testing	Training	Testing
Polynomial	0.0469	**0**	0.0764	0.0476	0.9120	0.9787
BP	0.4688	**0**	0.0799	0.1067	0.9052	0.8920
RBF	1.4844	**0**	**0.0762**	0.0467	**0.9125**	0.9819
ELM	**0.0156**	**0**	**0.0762**	**0.0456**	**0.9125**	**0.9818**
